# Management of surgical diseases of thyroid gland indications of the United Italian Society of Endocrine Surgery (SIUEC)

**DOI:** 10.1007/s13304-023-01522-7

**Published:** 2023-05-18

**Authors:** P. Del Rio, A. Polistena, M. G. Chiofalo, L. De Pasquale, G. Dionigi, G. Docimo, G. Graceffa, M. Iacobone, Fabio Medas, A. Pezzolla, S. Sorrenti, S. Spiezia, P. G. Calò

**Affiliations:** 1 Unit of General Surgery, Department of Medicine and Surgery, University of Parma, Parma, Italy; 2 Department of Surgery Pietro Valdoni, University of Rome Sapienza, Rome, Italy; 3 Department Head and Neck, Thyroid Surgery Unit, Istituto Nazionale Tumori IRCCS Fondazione G. Pascale, Naples, Italy; 4 Thyroid and Parathyroid Service, Otolaryngology Unit, ASST Santi Paolo e Carlo, Department of Health Sciences, University of Milan, Milan, Italy; 5 Department of Pathophysiology and Transplantation, University of Milan, Milan, Italy; 6 Division of Surgery, Istituto Auxologico Italiano Instituto di Ricovero e Cura a Carattere Scientifico (IRCCS), Milan, Italy; 7 Division of Thyroid Surgery, University of Campania “L. Vanvitelli”, Naples, Italy; 8 Department of Surgical Oncological and Oral Sciences, University of Palermo, Palermo, Italy; 9 Endocrine Surgery Unit, Department of Surgery, Oncology and Gastroenterology, Padova University Hospital, Padova, Italy; 10 Department of Surgical Sciences, University of Cagliari, Cagliari, Italy; 11 Division of Videolaparoscopic Surgery, Department of Emergency and Organ Transplanatation, University of Bari “A. Moro”, Bari, Italy; 12 Department of Surgery, “Sapienza” University of Rome, Rome, Italy; 13 Department of Endocrine and Ultrasound-guided Surgery, Ospedale del Mare, Naples, Italy

**Keywords:** Italian Society of Endocrine Surgery, Recommendations, Surgical diseases of thyroid gland, Thyroidectomy

## Abstract

A task force of the United Italian society of Endocrine Surgery (SIUEC) was commissioned to review the position statement on diagnostic, therapeutic and health‑care management protocol in thyroid surgery published in 2016, at the light of new technologies, recent oncological concepts, and tailored approaches. The objective of this publication was to support surgeons with modern rational protocols of treatment that can be shared by health-care professionals, taking into account important clinical, healthcare and therapeutic aspects, as well as potential sequelae and complications. The task force consists of 13 members of the SIUEC highly trained and experienced in thyroid surgery. The main topics concern clinical evaluation and preoperative workup, patient preparation for surgery, surgical treatment, non-surgical options, postoperative management, prevention and management of major complications, outpatient care and follow-up.

## Introduction

The United Italian Society of Endocrine Surgery (SIUEC) was established in Bari in 2017 from the union of two pre-existing societies (the Italian Society of Endocrine Surgery, SIEC and the Italian Association of Endocrine Surgery Units, U.E.C. CLUB). Following the long history and activities of the two founder societies, SIUEC promoted in the last 5-year several conferences, congresses, scientific publications and professional training activities. There is a specific need to define and promote among the entire endocrine surgical community in Italy updated recommendations for the diagnosis and management of surgical thyroid diseases, according to international protocols.

At the light of new technologies, recent oncological concepts, tailored approaches and based on the previous experience of the position statement on diagnostic, therapeutic and health‑care management protocol in thyroid surgery published in 2016 by the U.E.C. CLUB [1], an updated revision of the previous protocol was drafted by a SIUEC experts’ commission to provide clear clinical indications for endocrine surgeons in Italy. The objective of this publication is to support surgeons with modern rational protocols of treatment that can be shared by health-care professionals, taking into account important clinical, healthcare and therapeutic aspects, as well as potential sequelae and complications.

Comprehensive recommendations in the different clinical scenarios were provided.

However, it is not within the scope of the authors nor of the SIUEC to influence in any way the physician–patient relationship, which is based on trust and clinical judgment in each individual case.

Topics:

- SIUEC accreditation system for thyroid centers
- Clinical evaluation and preoperative workup;
- Hospital admission and waiting time;
- Therapeutic pathway:
  - patient preparation for surgery,
  - surgical and thermoablative treatment,
  - postoperative management,
  - prevention and management of major complications

- Hospital discharge and patient information;
- Outpatient care and follow-up.

### SIUEC accreditation system for thyroid centers

SIUEC has set up an accreditation system to verify the possession by each endocrine surgical unit of qualitative and quantitative requirements, and to stratify the centres regarding the level of competence. The following conditions are needed to obtain accreditation as thyroid centre: at least two surgeons devoted to thyroid surgery with appropriate training and number of thyroidectomies performed, routine application of international guidelines, at least 50 thyroidectomies performed each year, 24-h anaesthesiologic ward, accessibility to intensive care unit and cardiological intensive care unit, pathologist with experience in thyroid diseases, imaging (US, CT) available on a 24-h basis, presence of services of endocrinology, otolaryngology, nuclear medicine, vascular surgery in the same hospital or in a local or regional network. The centres with these requirements are defined as thyroid referral centres; in addition, centres performing ≥ 500 thyroidectomies per year are accredited as national thyroid referral centres.

## Clinical evaluation and pre-operative work up

A careful endocrinological evaluation, in terms of history, physical examination, laboratory and imaging, improves the surgical approach to thyroid disease facilitating the best tailored treatment. A multidisciplinary team (MDT) is advocate for thyroid disease management which includes specialists in: endocrine surgery, endocrinology, head and neck radiotherapy, oncology, nuclear medicine. MTD is required to provide individualized and optimal multimodality treatment for the initial evaluation and for further follow-up and treatments.

Medical history should be stored for previous malignancy, neck or whole-body irradiation for bone marrow transplantation, exposure to ionizing radiation during childhood, familial syndromes that include thyroid cancer (such as MEN 2, Werner Syndrome, Cowden Syndrome, familial polyposis, Carney complex, etc), clinical records as dysphonia, dysphagia, overgrowth of normal thyroid tissue. Physical examination of the neck includes an accurate inspection of the neck for any scars, mass or nodular enlargement; a complete palpation of thyroid gland between the cricoid cartilage and the suprasternal notch and palpation of the thyroid gland, the adjacent lymphatic chains, any present masses.

## Laboratory studies

### First-tier assessments

TSH serum level should be obtained for the first endocrinological evaluation [1–3] In case of a suppressed TSH (hyperthyroidism) FT3, FT4 and anti -TPO -TG -TSH receptor antibodies are required, 

In the instance of an elevated TSH (hypothyroidism) only antibodies anti TPO and anti TG are recommended.

- Serum calcium and phosphorus should be checked routinely.

### Second-tier assessments

Calcitonin (CT) is a reliable serum marker of medullary thyroid carcinoma (MCT) largely used for diagnosis and follow-up. Clearly (over cut-off) high serum values found in patients affected by undiagnosed thyroid nodules prevent the need of FNA for cytological assessment, often inconclusive or indeterminate, or for intralesional thyroid CT dosage. However, CT dosage remains essential in case of suspicious lymph node metastases, both in preoperative assessment and in case of suspected recurrent disease. The CT measurement provides an earlier diagnosis and allows the familial screening with the subsequent improvement of surgical outcome and patients survival. Borderline values may require a further evaluation to avoid false positive results, due to interference of drugs (as pomp inhibitors) or co-morbidity. In doubt cases, as well as for screening RET gene carriers and to differentiate between C-cell hyperplasia and medullary thyroid cancer [1, 3, 4] calcium gluconate stimulation test may be performed.

The routine measurement of calcitonin is still debated in literature because of the rare occurrence of CMT in front of the overall thyroid malignancies (5–10%) and in front moreover of the overall thyroid disorders, conditioning a relatively low CMT predictivity. It is actually recommended to measure the level of CT prior to thyroidectomy.

- *CEA* dosage is required in all patients with elevated CT, representing an unfavorable prognostic factor. Conversely, CT dosage is mandatory in patients presenting incidental high serum CEA.
- Serum calcium is useful for the screening of an incidental primary hyperparathyroidism [5].
- Serum phosphate and parathyroid hormone (PTH) In case of hypercalcemia [5].
- Anti-TPO (anti-thyroid peroxidase) and anti-Tg(anti-thyroglobulin) antibodies in case of suspected autoimmune disease [1, 4–6].
- TSH-receptor antibody (TRab) in case of suspected Grave’s Disease [1, 4–6].

## Instrumental studies

### First-tier assessments

- Thyroid ultrasound (US)with colour flow Doppler scanning represents an essential test in all patients with a clinical suspicion of thyroid nodule and/or nodular goitre, in all cases of incidental radiological finding of thyroid nodular disease (CT or MRI of the neck, thyroidal uptake on 18FDG- PET scan, etc.). Ultrasonography allows an accurate morphological evaluation of the thyroid and adjacent regional lymph nodes aimed at the acquisition of specific information regarding [1, 3, 5–7]:
  - location, size (possibly total gland volume) and structure of the lobes;
  - the presence, number, size and structure (solid, cystic, mixed) of the thyroid nodules;
  - vascular pattern of the nodule on colour Doppler;
  - status of the contralateral lobe in case of unilateral disease;
  - nodular features indicative of malignancy (hypoechogenicity, micro-calcifications, absence of halo, irregular margins, chaotic intranodular vascularity, round shape);
  - condition of the trachea (midline, displaced, compressed);
  - status of regional lymph nodes (reactive or suspicious).

The American College of Radiology Thyroid Imaging Reporting and Data Systems (TIRADS) is a 5-point classification to determine the risk of cancer in thyroid nodules based on ultrasound characteristics. This classification is actually largely used to an accurate study of patients affected by thyroid nodules [6].

### Second-tier assessments

Second-tier assessments are aimed at further diagnostic evaluation and at defining the therapeutic strategy, particularly in case of minimally invasive approaches and re-intervention:

- *Fine-needle aspiration cytology* (*FNAC*) is the most accurate and cost-effective method for evaluating thyroid nodules. Ultrasound guidance, cyto-assisted procedure (cytologist present), significantly reduces the number of non- diagnostic results and false negatives, especially in the case of nodules with a high likelihood of non-diagnostic cytology (> 25–50% cystic component) and/or difficult to palpate or posteriorly located nodules.

FNAC is indicated in all clinically or sonographically suspicious nodules with a diameter $\ge$ 1 cm. It is not recommended as a routine procedure for subcentimetric nodules; however, for nodules < 1 cm, FNAC is recommended in the following situations:

- family history of thyroid cancer,
- the presence of suspicious cervical lymphadenopathy,
- prior radiation therapy to the head, neck and/or mediastinum,
- exposure to ionizing radiation during childhood or adolescence,
- nodule with suspicious sonographic features (hypoechogenicity, microcalcifications, marked vascularity).

The cytology report should be descriptive, but also end with the assignment of the patient to a clearly defined and identifiable diagnostic category [1, 4–7].

After the publication of the Italian Societies of Endocrinology and the SIAPEC-IAP, in 2010–2014 [8, 9], the cytological classification is largely adopted (Table 1) and the retrospective cross-sectional survey, published in 2019, reports that the new SIAPEC-IAC criteria significantly increased the proportion of the overall TIR3 diagnosis. The division of TIR3 nodules into two subgroups (A and B) allowed a better evaluation of the oncologic risk and a better selection of patients for surgery [10]. In addition, if the cytological finding is uncertain, it is recommended to check the FNAC for BRAF V600E mutation.

- *Contrast-enhanced ultrasound (CEUS)* represents a non-invasive technique for the differential diagnosis of thyroid nodules. However, due to the operator-dependent evaluation, CEUS has rather variable sensitivity (68–100%) and specificity (67–94%) [11].
- *Thyroid* 99Tc-Pertenectate *scintigraphy* Indications for this investigation are limited to [12]:
  - subclinical or overt hyperthyroidism,
  - recurrent goiter,
  - suspicion of forgotten or ectopic goiter.

**Table 1:** The Italian thyroid cytology classification system (SIAPEC-IAP 2014) compared with the revised Bethesda reporting system (2017). The risk of malignancy of the revised Bethesda classification are reported considering the NIFTP as cancer

2014 Italian SIAPEC-IAP reporting system	Risk of malignancy (%)	2017 revised Bethesda reporting system for thyroid cytology
TIR 1 non-diagnostic TIR 1C non-diagnostic—cystic	Low	I. Non-diagnostic
TIR 2 benign	< 3	II. Benign
TIR 3A low-risk indeterminate lesion	10–30	III. Atypia of undetermined significance (AUS)/follicular lesion of undetermined significance (FLUS)
TIR 3B High-risk indeterminate lesion (oncocytic lesions included)	25–40	IV. Follicular neoplasm or suspicious for a follicular neoplasm (FN/SFN) (oncocytic lesion FNHCT/SFNHCT included)
TIR 4 suspicious of malignancy	50–75	V. Suspicious of malignancy
TIR 5 malignant	97–99	VI. Malignant

Radioactive iodine uptake test is reserved to patient proposed to 131- I streatment

- *Elastography* measures the degree of distortion of a tissue subjected to an external force and can therefore determine the elasticity of the tissue being examined. Malignant lesions often associate with changes in tissue mechanical properties; therefore, this technique may help refine the diagnosis of the lesion being examined. However, the applicability of elastography in clinical practice is limited by the variable sensitivity (54–69%) and specificity (60–96%) described in different reports [13].
- *Core needle biopsy (CNB)* Tissue biopsy is obtained by a cutting needle, usually equipped with a retractable spring-loaded mechanism (18–21 G Trucut needle). This method is carried out only under ultrasound guidance. The sampling of tissue that includes the periphery of the nodule and the surrounding thyroid parenchyma allows examining the architectural characteristics of the thyroid tissue, allowing a microhistological diagnosis. Recently, indications to CNB have been extended to nodules with inadequate (Tir 1) or indeterminate (Tir 3) cytology [12].
- *CT/MRI* These alternative imaging modalities may be useful in the assessment of large, rapidly growing, or retrosternal or invasive tumors to assess the involvement of extrathyroidal tissues [1, 4, 12, 14].
- *18F-FDG PET-CT* At present, 18F-FDG PET-CT cannot be considered a routine investigation [1, 4, 14] The procedure is indicated in re-staging patients presenting in the follow-up persistent or recurrent elevated serum thyroglobulin (DTC) or CMT calcitonin (CMT) levels due to local tumor persistence and/or distant progression/relapse. It is indicated also in preoperative staging of CMT, with diagnostic and prognostic value in association with DOPA-PET

This technique, to-day widely used by oncologists for the cancer staging, may incidentally reveal areas of increased uptake inside the thyroid gland, called incidentalomas”, which, in 25 % of cases, turn out to be cancer.

-* DOPA PET-CT is reserved to preoperative total body staging of CMT and to imaging the adrenal glands in hereditary MEN syndromes.

- *Laryngeal fibroscopy* Preoperative laryngoscopy is recommended in candidates to thyroidectomy to assess the morphological and functional integrity of the vocal folds. US evaluation of VC motility me be an alternative Mandatory indications are indicated as follows [1]:
- Patients complaining dysphonia
- Re-intervention (to exclude potential pre-existing injury of laryngeal nerves)
- Symptomatic large or substernal goiter
- Locally invasive thyroid cancer

## Hospital admission and waiting time

### Priority for hospital admission

In accordance with the Italian National Plan for the governing of waiting lists 2019–2021 [15], four priority classes for hospital admission are recognized for each pathology.

- Class A: hospitalization within thirty days for clinical cases that can potentially worsen rapidly to the point of becoming emerging or, in any case, seriously damaging the prognosis (All tumors, except Differentiated Thyroid Carcinoma < 1 cm limited to the gland without lymph node involvement, patients with severe thyrotoxicosis, patients with large goiter affecting significant stenosis of the airways)

- Class B: hospitalization within 60 days for clinical cases that present intense pain, or severe disability, and do not show a tendency to aggravate it nor can, due to the wait, seriously harm the prognosis (Differentiated Thyroid Carcinoma < 1 cm limited to the gland without lymph node involvement, Tir 3B and large Tir 3A nodules, hyperfunctioning disease without thyrotoxicosis, but with labile hormonal control with medical therapy)

- Class C: hospitalization within 180 days for clinical cases that present minimal pain, dysfunction or disability, and do not show a tendency to worsen nor can for the wait receive serious damage to the prognosis. (Hyperfunctioning goiter or Plummer adenoma with good hormonal control with medical therapy, goiter or cytologically benign nodules with a tendency to grow)

- Class D: hospitalization without maximum waiting time defined for clinical cases that do not cause any pain, dysfunction, or disability. However, these cases must be carried out at least within 12 months. (Large goiter stable with minimal growth at follow-up, goiters or Plummer with subclinical hyperthyroidism without the need of medical therapy)

### Pre-admission workup (or upon admission)

- Blood chemistry, complete blood count and coagulation tests for surgery;
- ECG;
- Chest X-ray (where indicated, depending on patient's age and comorbidities);
- Anesthesiology consultation;
- Vocal cord assessment.

### Recommendations for patients

- Patients should continue their current thyroid medications (methimazole, propylthiouracil, thyroxine, beta-blockers taken on a regular basis) until the day prior to surgery, unless otherwise indicated due to medical or anesthesiological reasons;
- Patients in treatment with vitamin K antagonist anticoagulants should discontinue therapy 3–5 days before surgery and should replace it with LMWH (last administration 24 h before surgery) [16];
- Patients in treatment with Non-vitamin K antagonist oral anticoagulants should discontinue therapy from 24 to 36 h before surgery to 1–2 days after surgery, without bridging them with LMWH [16–18];
- Patients should discontinue treatment with Clopidogrel or Ticagrelor 5 days before surgery, with Prasugrel 7 days before surgery, while Aspirin treatment does not need pre-operatory cessation [16].

### Admission

On the same day of surgery, unless otherwise indicated or required, but ensuring an appropriate timing for a detailed doctor-patient discussion about the surgical procedure and its risks, so that the patient can express his consent to the surgery in written documentation.

## Therapeutic pathway

### Patient preparation for surgery

- Antibiotics: antibiotic prophylaxis is not indicated in thyroidectomy [19, 20] except for particular cases, such as: severe diabetes, cardiac valvular disease, immune deficiency (hemodialysis or transplanted patients).
- Antithrombotic prophylaxis: international guidelines [19, 21] do not make specific recommendations regarding thyroid or neck surgery.
- Blood units: autologous pre-deposit blood donation or preparation of blood units for thyroidectomy is not justified.
- Position on the operating table (joint responsibility of the surgeon and anesthesiologist): patient in the supine position with a small wedge beneath the shoulders, at the scapular level, such to allow a mild hyperextension of the neck; with the neck in hyperextension, although mild, arms should be adducted and secured next to the patient's body in order to avoid rare, but severe and sometimes irreversible, brachial plexus paralyses due to stretch injury [22]; elbows should be adequately padded to avoid ulnar nerve paralysis secondary to compression; eye protection to avoid corneal ulceration and ocular trauma. When using intraoperative neuromonitoring, the endotracheal tube must be placed in a perfect position so that the surface electrodes adhere correctly to the vocal cords. Collaboration with the anesthetist is important.

### Informed consent

Patients should be adequately informed by the surgeon of the indications for surgery, possible alternative treatments, advantages expected from surgery, general and specific complications, rehabilitation therapy—if needed, and the clinical consequences of potential permanent postoperative injuries.

The information provided should be clearly explained, complete and prompt. After providing the most complete information, the physician will seek the patient’s consent to perform surgery, taking into full consideration any expression of dissent, even on individual aspects of the procedure or its potential consequences.

Transmission of information and the informed consent should preliminarily take place during the first visit and be renewed upon admission, before surgery, especially if enough time has passed such that the initial conditions may have changed. In fact, the patient must be given the opportunity to discuss in depth with his/her physician (or other trusted person) the information received and, if desired, to get information on the health facility where he or she will be treated and/or on the team that will perform the surgery.

Given the peculiarity of the therapeutic intervention (partial or total removal of the thyroid gland) and its potential consequences on the physical integrity of the subject [1] it is necessary that written documentation of the informed and conscious consent be retained, and that the informed consent process be documented in a specific chart note.

To this end, SIUEC is preparing a standardized consent form that will be made available on the website (https://siuec.it/).

### Surgical treatment

Of all the procedures that have been proposed for thyroid surgery, the following can be considered to be in current use [3, 19, 23–27]:

- Lobectomy plus isthmectomy
- Total thyroidectomy (considered as synonym for near-total thyroidectomy, as the presence of remnants, although minimal, is pretty much constant, without affecting the radicality of the intervention)
- Completion thyroidectomy after lobectomy

Beside classical surgical interventions, two new approaches must be introduced for the management of benign thyroid nodule and thyroid cancer in selected high-risk patients and of low-risk microcarcinoma:

- Percutaneous ablation (see appropriate section)
- Active surveillance (see appropriate section)

The surgical report must be accurate and provide a description of the thyroid gland and the macroscopic characteristics of the most relevant nodules, dimensions, inflammatory tissue, air way compression.

The surgeon must also report on the identification and preservation of the external branch of the superior laryngeal nerve, the identification of the inferior laryngeal nerves, the anatomical variations, mentioning dissection difficulties, if any [28–30]. The surgical report must highlight the side and number of parathyroid glands identified. A decision to leave macroscopic thyroid tissue in situ should be substantiated as the location and size. if neuromonitoring is used during surgery, the technique performed (intermittent or continuous) must be reported as the signal coded as per protocol. In case of Loss of signal we can choice the two-stage thyroidectomy; this should be accurately reported in the report. The use of the energy devices must also be reported in the report. (for neuromonitoring see appropriate section)

### Minimally invasive techniques

In the last few years, several techniques have been developed for minimally invasive thyroidectomy [31, 32]. Minimally invasive approaches for thyroidectomy can be classified into techniques with and without the use of endoscope.

The minimal incision thyroidectomy (MIT) differs from conventional thyroidectomy by a shorter skin incision and involve the use of optical aids (magnifying glasses 2.5 × 3.5) [31]. Techniques that involve the use of an endoscope can be divided into pure endoscopic techniques and video assisted techniques. An important limitation of endoscopic techniques is the difficulty of purely endoscopic dissection, especially when using accesses that are completely different from those used in conventional surgery (axillary, breast, chest access), which limited their application to the experiences reported by the authors who have proposed such approaches [31, 33]. Minimally invasive videoassisted thyroidectomy (MIVAT) is a totally gasless technique that involves a 1.5–2.0 cm central horizontal incision, use of a 5 mm 30° endoscope and dedicated dissection tools [34–36].

This technique was born more than 20 years ago, has large consensus and is safe and reproducible, with similar complication as compared to conventional surgery and significant advantages in terms of postoperative pain and discomfort, as well as of the aesthetic improved outcome and was supported over time by the use of increasingly performing laparoscopic columns in defining and magnifying the operating field [37–41].

Its wide diffusion allowed to collect data on its safety in the treatment of DTC with low–intermediate risk. Indications and controindications to MIVAT are reported in Table 2 [34, 35, 37, 38, 42–44]. Among the minimally invasive methods, the Trans-Oral Endoscopic Thyroidectomy Vestibular Approach (TOETVA) although largely reported in clinical studies, is still in a validation phase and requires a further confirmation on a larger clinical experience for a correct comparison not only of feasibility but also of complication rates [45, 46].

### Robotic thyroidectomy

The use of robotic technology has improved in the last years and allows, in the head and neck area, several indirect approaches for thyroidectomy: gasless transaxillary (the most used) [32, 47], transoral [48] and retroauricolar [49] and axillo-breast approach [50].

Indication and contraindications to robotic thyroidectomy [47, 51] are reported in Table 3.

**Table 2:** Indication and containdication to MIVAT

Indications	Contraindications	Relative contraindications
Thyroid nodules < 35 mm	Prior neck surgery	Prior neck irradiation
Thyroid volume < 30 ml	Locally advanced thyroid carcinoma	Thyroiditis or Graves disease
Low and intermediate risk papillary thyroid carcinoma	Preoperative evidence of lymphnode metastases	
Patients with RET gene mutation		

The use of transaxillary robotic approach to thyroidectomy is a feasible and safe option in selected patients who refuse incisions in the cervical region [32, 52, 53].

### Intraoperative nerve monitoring (IONM)

Injury to the inferior laryngeal nerve is one of the most feared complications following thyroidectomy, and medical litigation is becoming more frequent [19, 54–59].

In addition, it is well known that the anatomical integrity of nerves and thus not all nerve injuries are recognized intraoperatively [60–62].

Several techniques have been proposed for intraoperative monitoring of the recurrent laryngeal nerve (IONM). However, the most widely used and standardized method is the use of endotracheal tube surface electrodes placed in contact with the mucosa of the vocal cords [62, 63] Use of the IONM should also be indicated to identify the external branch of the superior laryngeal nerve (EBSN) [30].

The use of IONM during thyroidectomy (conventional-mini-invasive) may be associated with a significant reduction in the risk of bilateral vocal cord palsy if loss of signal to the inferior laryngeal nerve or vagus nerve is detected after the first lobectomy during total thyroidectomy [64–66].

It is of utmost importance to perform neuromonitoring according to the steps indicated in Table 4 [63]. More promising results are expected from continuous intraoperative nerve monitoring (C-IONM), which allows real-time assessment of inferior laryngeal nerve function during surgical manoeuvres and thus could prevent intraoperative injury [67].

**Table 3:** Indications and contraindications to robotic thyroidectomy

Indications	Contraindications	Relative contraindications
Benign disease:nodule < 5 cm	Previous thyroid or breast surgery	Arthrosis or previous surgery of the shoulder joint
Indeterminate nodules	Previous head and neck irradiation	Thyroiditis or Graves’ disease
Small differentiated thyroid carcinoma	Electronic medical devices (pacemakers,defibrillators in the pectoral region)	BMI > 30
		Distance from the axilla to the sternal notch > 15 cm

### Surgical treatment of hyperthyroidism

The most common causes include Graves’ disease (GD), toxic multinodular goiter (TMNG), and toxic adenoma (TA). Treatment strategy in hyperthyroidism firstly requires recovery of euthyroidism by antithyroid therapy; however, patients that not reach an euthyroidism state, even after antithyroid therapy, can be safely operated. Beta-adrenergic blockade is recommended in all patients with symptomatic thyrotoxicosis, especially in elderly patients. No particular treatment modality, among RAI, antithyroid medications or surgery, has demonstrated superiority over the others. Treatment modalities, pro-cons and side-effects need to be shared and discussed with the patient [68].

RAI is the most commonly used definitive treatment option for GD. Surgery offers a definitive treatment too and it is proposed as a first line treatment, primarily for patients with severe ophthalmopathy or goiter. Relapse rate is highest among patients receiving anti-thyroid drugs (approximately 40%), followed by RAI (21%), whereas surgery is associated to the lowest rate (<5%) [69].

Total thyroidectomy is more effective than subtotal thyroidectomy at preventing recurrent hyperthyroidism in Graves' disease and it is therefore recommended as the option of choice. Furthermore the type of surgery does not affect regression of Graves' ophthalmopathy [70]. If surgery is performed for all of the above conditions of hyperthyroidism, a preoperative treatment with methimazole must be administered; potassium iodide may be given in the immediate preoperative period in GD. Antithyroid drugs should be stopped at the time of thyroidectomy and beta-adrenergic blockers should be maintained and weaned following surgery.

Surgical options include:

- Isolated toxic adenoma (TA): an ipsilateral thyroid lobectomy, or isthmectomy if the adenoma is in the thyroid isthmus, should be performed for isolated TAs. Large nodules (≥ 3 cm), compressive symptoms and contraindication to RAI are specific indications to surgical treatment.
- Toxic multinodular goiter (TMG): near-total or total thyroidectomy are recommended.
- Flajani–Basedow–Graves’ disease (FBGD): near-total or total thyroidectomy are the procedures of choice (specific indication are the above mentioned for TA plus ineffectiveness or recurrence after thyrostatic treatment, young age and severe and ophthalmopathy).

Alternative therapies as thermal ablation of TA and TMNG can be considered in select patients in whom RAI, surgery, and long-term anti-thyroid drugs are inappropriate, contraindicated, or refused, but this approach obtained no recommendation at present [68].

### Surgical treatment of euthyroidism

Surgery is the only option that guarantees a definitive treatment with removal of the goiter despite the risk of specific complications. Main indications for surgery include compression symptoms, suspicion for malignancy, prevention of complication from progressive enlargement or mediastinal extension (with complications related to tracheal deviation and narrowing, superior vena cava syndrome) and occasionally for cosmesis. Surgery should also be considered in nodules that develop suspicious US changes or increase in volume and become symptomatic. The preferred extent of resection is lobectomy for benign uninodular goiter and total thyroidectomy for bilateral multinodular goiter.

- Unilateral nodular benign disease (negative cytology; normal contralateral lobe): lobectomy plus isthmectomy [3, 24]
- Unilateral nodular disease with cytological evidence of indeterminate nodules, with normal contralateral lobe: lobectomy and if available prior molecular testing [8, 71, 72]
  - Multinodular goiter: total thyroidectomy [3, 24]
- Asymptomatic nodules with modest growth and negative cytology and asymptomatic cystic nodules may be followed conservatively without intervention [3].

### Surgical treatment of substernal goiter

By definition, a substernal goiter extends into the mediastinum by at least 50% of its volume. The incidence is reported to be between 1 and 30%. It is classified as primary (very rare, originating from ectopic mediastinal thyroid tissue, with no connection to the cervical thyroid gland, and blood supply from branches of the aorta, the innominate artery or the internal mammary artery) or secondary (originating from the thyroid, with preserved vascular, parenchymal or fibrous connection to the gland) [73, 74].

Being most of the mediastinal goiter connected to the cervical thyroid gland, usual surgical approach consists in a total or near total, bilateral or mono-lobar retro-sternal thyroidectomy, according the bilateral or only mono-lobar thyroid involvment, through a cervical access, which is possible in 90 % of cases [73–75].

Additional sternotomy and/or thoracotomy are necessary for malignancies with local infiltration of the mediastinum, for rare cases of primary substernal goiter or when the mediastinal and/or retrovascular component of the goiter is such that to make removal through the cervical incision dangerous or impossible. Other factors favouring additional extra-cervical accesses include potential fibrosis from prior radiation or surgery; caudal extension below the arch of the aorta or involving the posterior mediastinum and ectopic mediastinal goiter not connected to the cervical gland [24, 76]. Surgical procedures are burdened with a greater incidence of complications due to complex vascular control and risk of haemorrhage, tracheal compression with risk of severe tracheomalacia and modification in normal anatomical landmark for inferior laryngeal nerves.

Neck CT scan imaging for suspected substernal goiter is recommended preoperatively to quantify the caudal extent with a 3D reconstruction and to confirm tracheal compression [24].

### Surgical treatment of thyroid malignancy

Adequate surgery is the most important treatment variable influencing prognosis. There is evidence to recommend total thyroidectomy in most cases, but lobectomy may be considered for intrathyroidal, uni-or multiple unilateral small tumors.

- Differentiated carcinoma: surgery should ensure removal of the primary tumor and of disease that has extended beyond the thyroid capsule, and clinically metastatic lymph nodes with contained incidence of postoperative complications. The extent of thyroidectomy (hemithyroidectomy versus total thyroidectomy) for differentiated thyroid carcinoma is still debated. Total thyroidectomy ensures good local control of cancer by removing even microscopic tumor foci (that are frequent and often bilateral), facilitates subsequent RAI treatment and allows adequate follow-up. Despite total thyroidectomy is still considered the standard of care for most of thyroid malignancy, there is no definitive evidence supporting a more aggressive surgical approach in terms of recurrence risk reduction and absolute survival [3, 23, 25, 77].

Due to the concern for over-treatment, surgical management of thyroid cancer has evolved in recent years. There is clear evidence supporting an appropriate risk assessment to recommend more aggressive interventions for high-risk patients and less aggressive therapies for low risk patients. The surgical treatment of the low-, intermediate-risk DTC (1–4 cm) remains still controversial. After careful patient selection hemithyroidectomy, in patients with DTC with diameter < 2 cm without other specific risk factors, could be a safe alternative [3, 78, 79].

Elderly or fragile patients might benefit of this tailored approach despite tumor risk factors must be always considered [80].

In the scenario of increased overall rate and early diagnosis of thyroid cancer, after accurate patient and tumor selection, recent evidence supports an active surveillance instead of up-front surgery in the management of low-risk thyroid microcarcinoma to avoid overtreatment and complications from unnecessary surgery [81–83].

For lymphadenectomy, please refer to the appropriate section.

- Medullary carcinoma
  Medullary thyroid carcinoma (MTC) is a rare neuroendocrine tumor originating from the parafollicular which presents in both sporadic (70 %) and hereditary (30 %) forms, either isolated or as part of a multiple endocrine neoplasia: MEN 2A or Sipple’s syndrome (in association with pheochromocytoma and hyperparathyroidism); MEN 2B or Gorlin–Steinert syndrome (in association with pheochromocytoma, ganglioneuromatosis and marfanoid habitus). Demonstration of calcitonin (CT) increased value is mandatory for the diagnosis. All patients with MTC should be offered genetic counselling due to the evidence that RET proto-oncogene mutations are detected in 90% of MTCs and are considered the predominant drivers of these tumours. RET mutations may occur both sporadically or can be inherited as germline events associated with familial MTC or with the multiple endocrine neoplasia syndromes.
  There is a strong recommendation for total thyroidectomy plus central node dissection, whatever values of CT are detected, being surgery the only potentially curative treatment. More extended lymphadenectomies are related to CT values and ultrasound findings (for lymphadenectomy, please refer to the appropriate section).
  In multiple endocrine neoplasia syndromes, a personalized strategy must be defined, in a multidisciplinary setting, to plan the priority of treatment of the associated tumors, mostly considering the concomitance of a pheocromocytoma and the age of the patient. Usually, adrenalectomy must be performed prior to thyroidectomy, and parathyroidectomy during thyroidectomy [1, 25, 84].

- Undifferentiated or anaplastic carcinoma
  Undifferentiated or anaplastic carcinoma is a rare thyroid tumor associated to poor prognosis in most cases (median survival of approximately 5 months). Advanced disease with extensive local infiltration and/or distant metastases are typically detected at the clinical onset. Approximately 10% of patients with ATC present with only an intrathyroidal tumor, whereas 40% have extrathyroidal invasion and/or lymph node metastasis, with the remainder of patients presenting with widely metastatic disease [25, 85].
  When anaplastic carcinoma is suspected, clinical and instrumental assessments of disease by a multidisciplinary team (surgeon, endocrinologist, pathologist, oncologist, radiation oncologist, radiologist) are pivotal for the evaluation of treatment options and advantages/disadvantages of the proposed treatments which must always be discussed with the patient with a clear focus to the patient’s goals of care and life expectancy [25, 85].
  Endoscopic evaluation (both fibreoptic laryngotracheoscopy and bronchoscopy, often also esophagoscopy and if indicated endoscopic ultrasonography) and imaging with CT scan or MRI with contrast and PET scan are fundamental for a precise assessment of local invasiveness and to exclude the presence of distant metastasis. Evaluation of airway status is of primary importance especially in order to schedule palliative treatment for this life-threatening complication.
  The surgical treatment can achieve three possible options: palliation, prevention of future complications or curative intent. The option of neoadjuvant and/or adjuvant therapy must affect the decision for a possible surgical resection and the approaches and treatments must be always planned as multimodal [25, 85].
  All anaplastic thyroid cancers are considered as stage IV (AJCC/UICC): stage IVa and IVb patients may be potential candidates for a multimodal treatment including a more or less radical surgical resection associated with radio- and chemotherapy, which, in some cases, allows subsequent surgical re-exploration for local disease control. In patients with stage IVc, treatment options remain limited and controversial. Treatment is mostly palliative (tracheostomy or tracheal esophageal stent), with the intent of improving patients’ quality of life as much as possible and guarantee sustainable end-of-life conditions according to the patient’s preferences.
  Surgical resection with curative intent should be offered to patients with loco-regional disease only if complete tumor resection can be achieved with minimal morbidity being R0/R1 resections independently associated with longer overall survival.

Surgical treatment usually consists in [25, 85]:

- a total thyroidectomy or a near-total thyroidectomy (48.6%) with associated lymphadenectomy, with only a limited group of patients (1.7%) receiving a larygectomy/pharyngectomy as part of the procedure.
- The role of thyroid lobectomy is still debated with insufficient data regarding the clinical outcome. This option can be only considered in patients with localized and resectable ATC (normal contralateral lobe on preoperative ultrasound) and documented injury to the ipsilateral recurrent laryngeal nerve, or no identification of the ipsilateral parathyroid glands.
  - ATC extensively involving the upper aero-digestive tract or major vascular or mediastinal structures, is generally not considered for surgical resection. However, tailored treatment should be considered on individual basis when radical resection with appropriate reconstruction can be achieved supported by expected benefit from associated treatments such as chemoradiation. However, it must be considered that surgical intervention may delay external beam radiation or systemic chemotherapy because of wound complications.
  - Pre-emptive tracheostomy placement is not recommended in patients without impending airway compromise. It is instead recommended in case of symptomatic patients or as prevention of laryngeal and tracheal edema before radiotherapy.
  - For incidental ATC limited to the thyroid, a total thyroidectomy is the appropriate treatment and either lobectomy can be considered since there are no data that demonstrate a difference in disease free survival or survival based on the extent of thyroidectomy.

**Table 4:** IONM Standards steps.

L1: vocal cord examination with preoperative laryngoscopy
V1: stimulation of the ipsilateral vagus before RLN dissection
R1:stimulation of the RLN at the first point where it is found in the tracheoesophageal groove
S1: stimulation of the EBSLN with the probe after it has been detected
S2: stimulation of the EBSLN proximal to the point where superior thyroid vessels are separated,following separation of the vessels and successful bleeding control
R2: stimulation of the RLN at its most proximal point after the dissection is complete
V2: vagus stimulation after bleeding control is complete at the surgical field
L2: vocal cord examination with postoperative laryngoscopy

For lymphadenectomy, please refer to the appropriate section.

### Thermoablative treatment

Ultrasound-guided thermoablative treatment (TA) procedures are used as alternative treatment in selected clinical cases. The international guidelines have standardized their use [86, 87]. TA procedures are well tolerated by the patient but require specific operator training. The TAs are radiofrequency ablation (RFA), laser ablation (LA), microwave (MWA) and high intensity focused ultrasound TA (HIFU). Symptomatic benign nodules, hyperfunctioning nodules, microcarcinomas and recurrent lymph node metastases can be treated [88–94]. TA treatments induce a reduction in nodular volume and an improvement in symptoms. Over the years it is possible to have a volumetric increase in the lump that may require re-treatment or surgery [95, 96]. LA and RFA are currently the most used techniques, whereas MWA and HIFU are still under study.

### Selection criteria for patients with benign symptomatic nodular lesion

Symptoms are compression, cosmetic damage, and tracheal deviation. Before TA, patients will have to perform two FNABs of the lesion with benign outcome (TIR2/Thy2) [86, 87, 97]. Symptoms are influenced by the location of the lesion, the circumference of the neck and other factors that cannot be objectively assessed [97]. Therefore, according to the international guidelines [86, 97], the TA of benign nodules should be considered for patients with nodules with a diameter ≥ 30 mm or with a minimum volume of 6 ml who complain of local discomfort. Treatment of asymptomatic lesions is not recommended.

### Selection criteria for patients with compressive multinodular goiter

In patients with compressive multinodular goiter, TC or MRI should be performed and TA should be limited to cases with a dominant nodule not suitable for surgery due to concomitant diseases or at the explicit request of the patient [86, 98].

### Selection criteria for patients with hyperfunctioning nodule

In hyperfunctioning thyroid nodule, a 99Tc-scintigraphy should be performed. In Plummer's adenoma less than 10 ml the patient can be subjected to TA [3, 86].

### Selection criteria for patients with microcarcinoma

Literature suggests active surveillance of microcarcinomas only in selected patients [3]. A valid alternative is TA in all those patients who refuse surgery and who experience active surveillance with anxiety and concern [99]. Patients undergoing TA showed a significant reduction in lesion volume, negative cytology for malignant cells at follow up and no statistically significant difference in the risk of lymph node recurrence compared to the surgery group [99–104].

### Selection criteria for patients with lymph node recurrence from papillary carcinoma

Lymph node recurrences are treated with surgical excision, metabolic radiotherapy, TA and thyroid suppressive hormone therapy [98–104]. TA can be used in the treatment of lymph node recurrences in patients who refuse surgery or who suffer from comorbidities such that surgery is not recommended [87, 96, 97].

Localized treatment with TA is useful in patients with a single metastasis, in those with metastases at high risk of local complications and should be performed in patients prior to the initiation of any systemic treatment [3]. In case of ultrasound suspicion, an FNAB with dosage of Thyroglobulin in the eluate should be performed [3]. In addition, a contrast spiral CT and an 18FDG-PET/CT should also be performed to allow for adequate mapping, preoperative localization and staging [3, 104]. Treatment of single lymph node recurrences with TA resulted in a 55–95% reduction in lesion volume and complete disappearance of metastatic foci in 40–60% of cases [3, 104]. TA can be considered a valid alternative in high-risk surgical patients or those who refuse surgery [3].

### Regional neck nodes dissection

#### Differentiated thyroid cancer

Regional nodal disease is reported in 30–60% of patients with Papillary Thyroid Cancer (PTC) at diagnosis, even in clinically node-negative (cN0) patients.

All the patients undergoing surgery for thyroid cancer should undergo a thorough preoperative assessment of cervical lymph nodes, including central and lateral compartments. High-resolution ultrasound is sensitive and specific in detecting lymph node metastases and may identify non palpable lymph node metastases in up to a third of patients with papillary thyroid cancer.

The neck computed tomography or magnetic resonance imaging is not part of the routine initial evaluation. Still, it is recommended for patients with suspected advanced disease, especially in extensive lateral neck disease and for areas not accessible by ultrasound [105–109].

The most common site of lymph node metastasis from PTC is the central compartment, followed by the jugular chain [109].

Prophylactic central lymph node dissection (PCLND) in clinically node-negative (cN0) PTC patients is still a controversial issue [108, 109].

The potential benefits of (PCLND) include [27]:

- Reducing the risk of central neck recurrence.
- Increasing the accuracy of pathologic staging to guide adjuvant RAI therapy.
- Improving the accuracy of thyroglobulin surveillance during long-term follow-up.

It has been demonstrated that PCLND may increase the rate of complications, including hypoparathyroidism and recurrent laryngeal nerve (RLN) injury. In contrast, no high-level scientific evidence confirms a significant impact on the oncologic outcome [1, 109–113].

To date, PCLND in PTC is not routinely indicated [26, 114].

PCLND should be considered for PTC patients who present with advanced tumours (T3 and T4), bilateral or multifocal tumours, or known lateral cervical neck disease [3, 115].

Recently a more limited PCND, including removal of pre-laryngeal, pretracheal and paratracheal nodes on the side of the tumour, was proposed in selected patients with clinically unilateral DTC [116]. For PTC patients presenting with clinically involved lymph nodes in the central neck (cN1), therapeutic central neck dissection is recommended [117–121].

The central compartment includes level VI and VII lymph nodes. In level VI, the anatomic boundaries are the hyoid bone (superior), common carotid arteries (laterally), sternal notch (inferior), and prevertebral fascia (posterior). Level VII include the lymph nodes below level VI and are bordered by the innominate (brachiocephalic) artery.

Nodal metastases of the central compartment involve most frequently four major nodal basins: prelaryngeal (Delphian), left paratracheal, right paratracheal, and pretracheal. Out of there, parapharyngeal, retropharyngeal, retro oesophagal and upper mediastinal areas are more rarely involved.

The central neck dissection includes a comprehensive and compartmental dissection and may be unilateral or bilateral. Unilateral central neck dissection involves completely removing unilateral paratracheal nodes and pretracheal and prelaryngeal lymph nodes. Bilateral dissection includes the removal of pretracheal, prelaryngeal, and bilateral paratracheal lymph nodes.

Node picking of only the macroscopically involved nodes is not recommended [122–125].

#### Lateral neck

Clinically apparent lymph node metastases to the lateral neck are present in approximately 20–30% of patients at presentation.

Metastatic spread is mainly observed in levels III, IV, IIA, and VB and occasionally in the IIB and VA [126]. Skip metastasis to the lateral neck without central compartment can be seen approximately in 8.7–21.8% of patients [127–129].

Optimal treatment of patients with metastatic thyroid cancer requires the removal of macroscopic clinical cervical lymph node metastases at the time of initial surgery.

Therapeutic lateral neck dissection is recommended for patients with lateral lymph node metastasis proven by biopsy and/or thyroglobulin washout. Prophylactic lateral ND is not indicated for PTC.

The lateral neck dissection should be a compartment-based selective neck dissection of the levels IIA, III, IV, and V. Comprehensive clearance of these levels is associated with a lower risk of recurrence [130, 131]. To reduce the risk of spinal nerve injury and due to the low likelihood of lymph node involvement, the level IIB dissection is best avoided unless there are suspicious lymph nodes at level IIB or bulky disease at level IIA. Similarly, routine dissection of level VA is generally unnecessary and is only dissected when there are clinically apparent lymph node metastases [106, 130]. More selective lymph node dissection, such as levels III, IV or levels IIA, III, IV, may be considered in patients with well-differentiated thyroid carcinoma with limited lateral neck lymph node metastases without other risk factors [132].

Lateral ND can be associated with significant complications. The most frequent permanent complication is paresthesia of the lateral neck and ear, which results from injury to the greater auricular nerve and sensory cervical root branches. Up to 11% of patients undergoing lateral neck dissection develop chronic neck pain that can be secondary to injury of the accessory nerve or the cervical root branches during dissection in the posterior triangle. Temporary or permanent injury to the eleventh cranial nerve (accessory spinal nerve) can occur in 6% to 20% of cases, resulting in shoulder drop and inability to raise the arm. This risk is increased when levels IIB and VA are dissected. Less common complications of lateral neck dissection include injury to the phrenic nerve and the sympathetic chain, and these occur in less than 1% of patients [59, 133].

#### Medullary thyroid carcinoma

The initial surgical approach depends on preoperative serum calcitonin levels and neck imaging findings.

In patients with medullary cancer, sporadic or familial, without evidence of nodal disease, total thyroidectomy with a prophylactic bilateral central neck dissection at the initial operation is the standard treatment [25–84, 134].

Preoperative screening for pheochromocytoma and hyperparathyroidism is recommended for all patients with MTC [133].

Initial management of the image negative lateral neck compartments in MTC patients remains controversial.

The central neck nodal status and preoperative calcitonin levels may predict the probability of lateral neck involvement and guide treatment of the lateral neck compartments [135, 136].

In patients with medullary thyroid cancer and no evidence of neck metastases on ultrasonography and no distant metastases, lateral neck dissection (levels II–V) may be considered based on serum preoperative calcitonin levels. Elective ipsilateral lateral neck dissection might be performed if the basal calcitonin is above 200 pg/ml and if calcitonin levels are greater than 400 pg/ml, even contralateral lateral neck dissection has been proposed. However, this topic is still debated and these recommendations are not practised routinely by most surgeons [84, 137].

In conclusion, based on current practice guidelines and available evidence patients with MTC metastatic to the cervical lymph nodes should have a total thyroidectomy, dissection of the central compartment and comprehensive dissection of the involved lateral neck compartments (levels II–V).

When only the ipsilateral lateral neck compartment is positive at preoperative workup, contralateral neck dissection is recommended if the basal serum calcitonin level is greater than 200 pg/mL.

Lateral neck dissection, when indicated, should be comprehensive, including levels from II to V [25, 84].

### Anaplastic thyroid cancer

The incidence of neck lymph node metastases is high in anaplastic thyroid carcinoma. Lateral neck dissection should include levels II–V with central compartment clearance when surgery is performed with radical intent [1, 137].

## Postoperative complications and their management

For a correct management of patients undergoing thyroid surgery, it is essential that medical and nursing staff know all post-thyroidectomy complications and time of onset, to promptly treat especially those that can endanger patient’s life, such as haemorrhage, with compressive hematoma and bilateral paralysis of recurrent laryngeal nerve, with acute respiratory failure. Another complication that must be recognized quickly, is hypocalcemia, because, if severe, it can cause tetanic crisis.

Complications that do not immediately endanger patient's life are unilateral recurrent laryngeal nerve paralysis and External Branch of the Superior Laryngeal Nerve (EBSLN) injury.

### Prevention and management of complications haemorrhage with compressive hematoma

Post-thyroidectomy haemorrhage with compressive hematoma is a very serious complication that can lead to death of the patient [19]. It occurs at a rate ranging from 0.1 to 2% of patients, in a quarter of cases with the need to reopen the wound at the patient’s bed. Almost all cases occur in the first 6 h after the intervention, about 20% between 6 and 24 h postoperatively, and only in a few anecdotal cases after 24 h [138, 139].

Risk factors for bleeding have been identified, some related to the patient, others to the disease, others to the intervention and others to the surgeon. Those related to the patient, concern coagulation pathologies (such as hemophilia, liver diseases, chronic renal failure, etc), the intake of anticoagulant and antiplatelets drugs and the sex. The male sex was reported by some Authors as at greater risk than the female one [138]. About disease at greater risk of post-thyroidectomy bleeding, Graves’s, substernal and recurrent goitre have been reported [56, 140].

As regards the intervention, hemithyroidectomy is at higher risk than total thyroidectomy [139]. Risk factors related to the surgeon are strictly dependent on his experience: the greater the number of thyroidectomies performed, the less the risk of bleeding [138].

Fundamental aspect to prevent bleeding is an accurate and precise hemostasis technique. Hemostasis can be obtained in different ways. The conventional technique consists in the ligation/clamp and section of the vessels, with or without cautery. About cautery it may be used for small vessels. It is better to use a bipolar than a monopolar one, because the first prevents heat loss, avoiding adjacent structures such as parathyroids and inferior recurrent laryngeal nerve injuries. “Energy devices', so defined because they use different forms of energy such as radiofrequency or ultrasound or hybrid systems that combine both energy modalities, have proved to be very useful for thyroid surgery, without an increased risk of postoperative bleeding [141, 142]. As well as an accurate hemostasis, to reduce the risk of bleeding, the execution of a manoeuvre by the Anaesthetist that simulates a “Valsalva”, may help to highlight a small venous bleeding. Some Authors also suggest not to completely close pre-thyroid muscles and to leave an opening, to facilitate spontaneous decompression in case of bleeding [19]. The routine use of drain is not recommended. Its use should be considered on a case basis. Recent meta-analyses have shown that the use of suction drains does not prevent neck haematoma, and that, conversely, the postoperative stay is longer, and that the incidence of wound infection is higher [143, 144]. In addition, the use of local haemostatics is still controversial, but considering the findings currently available from two meta-analyses, their use has not shown advantages in reducing the rate of clinically relevant bleeding, while increasing the rate of wound infection [145, 146].

If hemorrhage with compressive hematoma develops, it must be diagnosed quickly. For this reason, the nursing staff must check the neck and dressing frequently in the first 24 postoperative hours, with particular attention to the appearance of neck swelling and, if present, to the drain. This must not be empty, because it could be blocked by clots, or full, as it indicates excessive blood loss. The nurse must alert the surgeon in case of neck swelling, exceeding blood loss from the drain, onset of restlessness, and dyspnea. In case of symptomatic hematoma, immediate reopening of the wound, in severe cases even bedside and before reintubation, quick reintubation, and surgical revision are necessary. The removal of clots and the revision of hemostasis are the definitive treatment for progressive and symptomatic hematoma. The observation is possible only in the case of small self-limiting hematomas.

### Hypoparathyroidism

Hypoparathyroidism is the most frequent complication after bilateral thyroid surgical interventions and consists in decreasing both parathyroid hormone (PTH) and calcium serum levels, below the lower reference value. This complication may be transient or definitive, depending on whether it resolves within 6 months or not [147].

The incidence of post-thyroidectomy hypoparathyroidism is variable in literature, ranging from 19 to 39% the transient and from 0 to 15% the definitive [148].

The main cause of hypoparathyroidism is the trauma caused by surgery, which can lead to devascularization of the parathyroid glands or to accidental removal of one or more parathyroids, from 3.8 to 9.7 % in literature [149, 150] A long duration of the surgical procedure and hypothermia may be further factors favouring this complication.

In addition to surgery, other risk factors for hypoparathyroidism linked to the disease and to the patient have been identified, such as Graves’, thyroiditis, retrosternal goiter, cancer with need of central neck dissection and the female sex.

A frequent condition is hypocalcemia with PTH values in the normal range. In these cases, metabolic factors, such as vitamin D deficiency and hyperthyroidism, have been implicated to explain the lowering of calcium levels, despite the normal PTH [151].

Surgeon’s experience minimises the risk of hypocalcemia, favouring the recognition of parathyroid glands, that could be confused and consequently damaged, with fat, thyroid nodules, and lymph nodes. A precise surgical technique consists in dissection of parathyroid glands, remaining as close as possible to the thyroid capsule, to preserve their vascularization.

This may be improved using loop magnification (4×). The use of auto- and immune-fluorescence methods has been proposed by several Authors both to better identify parathyroid during surgery and to predict postoperative hypoparathyroidism [152, 153] even if not all authors agree on the real usefulness of the procedure [154]. In case of venous congestion of a parathyroid gland, it would be advisable, to make incisions on the capsule, to facilitate blood outflow. When a parathyroid is accidentally removed, it is indicated to re-implant it, reduced in small fragments of 0.5–1 mm, in pockets of ipsilateral sternocleidomastoid muscle, after extemporaneous histological examination [19, 155].

Near-infrared autofluorescence (NIRAF) imaging is a promising technique and should be employed when available. This technique exploits the intrinsic fluorescence produced by parathyroid glands when exposed to a source light with a specific wavelength (820–830 nm) without the use of a contrast agent. Different studies have reported a significant decrease in both transient and definitive post-operative hypoparathyroidism [156, 157].

The lowest calcemia values are usually observed 24–72 h after thyroidectomy. Several methods have been proposed to predict early, within the first postoperative day, which patients will develop hypocalcemia. The combined dosage of post-operative PTH and serum calcium levels proved to be valid in the decision of which patients to treat [158, 159].

In case of hypocalcemia (calcium < 8 mg/dL) with normal PTH the patient can be treated with calcium carbonate at a variable dosage from 2 to 6 g per day, in at least three administrations, or the equivalent of Calcium citrate. When hypocalcemia is associated with low levels of PTH, in addition to oral calcium supplementation, it is also necessary to administer calcitriol at a variable dosage from 0.25 to 0.5 mcg, once or twice a day. In the event of severe symptomatic or asymptomatic hypocalcemia with calcium serum levels < 7.0–7.5 mg/dL, or drop in calcium levels despite oral calcium support, intravenous calcium is required: 2 g of Calcium gluconate diluted in 250 ml of normal saline, once or twice a day [158]. In these cases, it is also important to measure magnesium levels, because magnesium depletion reduces PTH secretion and activity. If it is < 1.6 mg/dL, in patients with normal renal function, it is necessary to supplement it with 400 mg of Mg oxide once or twice daily Levothyroxine should be taken one hour before or three hours after calcium supplementation [160, 161].

### Temporary or permanent damage to the recurrent laryngeal nerve and the external branch of the superior laryngeal nerve

Unilateral paralysis of the vocal cords (VC) as a result of unilateral paresis of the recurrent laryngeal nerve (RLN) can lead to a significant reduction in quality of life with dysphonia and dysphagia, which has consequences especially for young patients. In addition, shortness of breath may occur during speech due to uncontrolled air leakage. While improvement of the voice can be achieved with speech therapy in unilateral VC paresis, patients with bilateral RLN paresis are significantly restricted in their daily life and are disabled in the long term.

Bilateral RLN palsy is often associated with respiratory distress syndrome and may require permanent tracheotomy or glottis dilating surgery [62]. The risk of tracheostomy for bilateral RLN palsy averages 30%, with another 21% requiring other acute airway interventions. Thus, overall, 50% of patients with bilateral RLN palsy require airway intervention.

The true incidence of postoperative transient RLN complications appears to be approximately 3% to 12%. The incidence of long-term RLN complications such as permanent morbidity remains unknown. The same is true for the incidence of bilateral RLN palsy, whether transient or permanent, is unknown.

RLN palsy leads the world in the incidence of forensic litigation after thyroid surgery [62].

Pre- and postoperative laryngeal examination is a gold standard for RLN management, along with the surgeon's experience, training, routine exposure, identification of the nerve, and perfect knowledge of the surgical anatomy of the RLN.

For embryological reasons, the anatomical course of the right and left RLN differs. The right RLN is shorter because it winds around the (right-sided) brachiocephalic truncus, and the left RLN is longer because it winds around the dorsal aortic arch. Because of the aforementioned embryologic-anatomic features, the right RLN runs obliquely from lateral caudal to mediocranial, whereas the left RLN runs more vertically and parallel to the esophagotracheal axis. The different, embryologically determined nerve anatomy on both sides is also reflected in the different frequency of the following anatomic features and anomalies

#### Tuberculum Zuckerkandl

Tuberculum Zuckerkandl, seen to varying degrees in approximately 60% of thyroidectomies, is more common on the right side than on the left side above [62]. The RLN usually runs dorsal to the tubercle of Zuckerkandl, but it may cross the tubercle or only the anterior branch of the nerve crosses the tubercle.

#### Non-RLN

A non-RLN is found in about 1.5% of thyroid surgeries and, for embryological reasons, is almost always on the right side, and only extremely rarely (in the case of complete situs inversus) on the left side.

In addition to these two atypical nerve courses, which occur with varying frequency on both sides, there are variants of the RLN course on both sides related to the following anatomic structures and features of thyroid pathology:

#### Relationship between RLN and inferior thyroid artery (ITA)

In the area where the ITA crosses with the RLN, the RLN comes in close proximity to the thyroid gland. More than 20 positional variants of the RLN in relation to the upper and lower branches of the ITA have been described. There are three main variants that can be considered: the retrovascular, antevascular, and intervascular courses. An antevascular course is more commonly observed on the right side, and a retrovascular course is more commonly observed on the left side. When a CT-scan is available, a careful evaluation for an aberrant right sub-clavian artery (arteria lusoria) should be carried out to evaluate the presence of a non-RLN.

#### Ligamentum berry

The closest positional relationship between the RLN and the thyroid organ capsule is in the region of the ligament of Berry. Both intraligamentous and intrathyroidal location of the RLN may occur.

#### Extralaryngeal branches

Based on anatomic studies, extralaryngeal branches of the RLN occur in approximately 60–90% of nerve courses; there are usually 2, rarely 3, branches of the RLN, which usually arise from the main trunk above the junction and divide before entering the larynx. Isolated paralysis of the dorsal or ventral ramus is extremely important for surgery because either closure (ventral branch) or opening of the glottis (dorsal branch) is compromised.

#### Preoperatively predictable RLN risk

Anatomic criteria for determining patients at high risk for thyroid surgery have been described. Increased risk for RLN palsy is detectable preoperatively when locally advanced cancer is diagnosed or a large retrosternal goiter is detected on imaging. Graves' disease, Hashimoto's disease, de Quervain's disease, or Riedel's thyroiditis also indicate a potentially increased risk of RLN palsy. The risk of bilateral paralysis is particularly high in the presence of preexisting unilateral paralysis.

#### Preoperatively unpredictable RLN risk

Preoperatively unpredictable risk situations that can only be detected intraoperatively are one of the main reasons for using neuromonitoring (IONM) not only selectively but also routinely. Examples of intraoperative risk situations are: (1) atypical RLN pattern anterior to the thyroid gland; (2) RLN anterior to Zuckerkandl's node; (3) fixed, spread, or trapped RLN with capsular association through fascial bands; (4) vascular or goitrous lesions; (5) invaded RLN; (6) posterior ligament of Berry's nerve; (7) thin RLN (< 1 mm); (8) Branching of the antevascular RLN; or (9) non-RLN, which occurs in approximately 1.5% of thyroid surgeries. Atypical courses of RLN are observed in approximately one quarter of thyroid surgeries.

Finally, with the increasing use of IONM, there seems to be a need to adjust the resection strategy in case of intraoperative loss of signal (LOS) of the first operated side in planned total thyroidectomy. If a true LOS is confirmed, identification of the site of the lesion, i.e., mapping of the neural injury point, is recommended and then consideration of the optimal contralateral surgical timing is suggested. The option to abort surgery is suggested.

In addition to knowing normal anatomy and anatomical variants of the RLN, to prevent injuries, some technical measures are important. First is the visualisation of the nerve along the entire course in the operating field, up to the entrance into the larynx. It is important to keep the operating field bloodless, to avoid excessive traction on the nerve and to avoid the use of heat sources, such as cautery and energy-based devices, near it. In the case of RLN section, recognized intraoperatively, immediate repair is possible*.* The reconstruction does not allow the resumption of the motility of the vocal cord motility, but it seems to improve its tone and to facilitate the resumption of the voice, avoiding aspiration. There are several possibilities for nerve reconstruction and consist of microsurgery techniques. RLN reconstruction may be direct through a simple end-to-end fascicular anastomosis, if the lesion may be repaired without tension and the damaged section is less than 5 mm. When the section is more than 5 mm, anastomosis between the injured nerve and the ansa cervicalis nerve is considered the most suitable method. Direct reinnervation of thyrohyoid muscle has shown good results. Injection laryngoplasty with different materials, is a procedure with the aim of improving glottal closure and reducing the space between the paralyzed vocal fold and the normal. In all cases speech therapy is the first measure to be taken to restore the voice [162, 163].

In case of bilateral RLN injury and consequent bilateral vocal cord paralysis, at the time of extubation, the patient may develop acute respiratory failure, if the paralysis is in adduction. This situation requires reintubation and immediate tracheostomy should be avoided. The patient should be kept intubated for 24 h. After 24 h an extubation attempt under fibroscopic control should be made, with evaluation of the motility of the vocal folds. If the bilateral in adduction paralysis persists, another 24 hours of intubation are indicated. The tracheostomy is indicated if the condition with respiratory distress persists further. The prolonged safe extubation approach gives the possibility to reduce the need for tracheostomy up to 85%v of cases [164].

In case of definitive paralysis, various procedures have been proposed, and one of the most used is posterior laser cordectomy [165].

In case of bilateral vocal fold paralysis in paramedian position, with adequate respiratory space, tracheostomy is not necessary. Bilateral paralysis of the vocal cords, especially in this situation, is associated with swallowing disorders, with risk of inhalation. The use of thickening substances to liquids can limit this risk. Damage to the external branch of the superior laryngeal nerve (EBSLN) causes changes in pitch and vocal range. Identification of this nerve can be difficult. The technical trick of ligating the branches of the superior thyroid artery near the capsule, with slight lateral and caudal traction of the upper pole, may reduce the risk of nerve injury. Intraoperative neuromonitoring with stimulation and detection of the EBSLN before upper pole dissection may allow a higher rate of nerve preservation [30].

## Hospital discharge and patient information

It is recommended to inform patients of their expected date of discharge, 24-h in advance. On the expected day, and after checking the normality of the blood pressure, wound care, with no symptoms in the neck, bulging, or cervical bruising, and laboratory assessment of parathyroid function, instructions on how to care at home are provided, as well as how to control pain, ideally with no more than three analgesics [166].

### Short-day surgery criteria

Day hospital discharge has been described since 1980s and proved to be safe, with high patient satisfaction rate in selected cases [167].

General considerations to be applied for short day surgery are listed below, and require a standardized preoperative selection, and interdisciplinary collaboration between the surgeon, anaesthetist and nursing staff:

I. patients need to understand the implications of surgery and the planned follow up;
II. patients need adult accompaniment at their residence;
III. patients should stay close to the hospital facilities easily accessible within a maximum of 1 h in case of urgency;
IV. patients should be able to eat, without nausea, vomiting, or other adverse reactions;
V. patients should have been sat on a chair;
VI. patients voided.

According to the common experience in the affiliated Italian endocrine surgical units, the SIUEC experts’ panel recommend against short stay modality in whatever thyroid surgery.

### Hospital discharge summary

A comprehensive clinical report, with a copy included in the patient’s chart, should be given to the patient on the discharge day and addressed to the primary care physician. The attending physician who discharges the patient on the day should sign the report, containing also direct phone number of the hospital and clinic.

Recommended information to be included in the discharge summary are listed below:

1. date of admission and first diagnosis;
2. main tests performed during the hospital stay, focusing on those with an altered result and those requiring further investigation;
3. summary of the operative note, reporting the day and the type of surgery, any intraoperative difficulties encountered and, if performed, reimplantation of parathyroid tissue; detailed information on drains coming from the incision, as well as the type of wound closure (stitches etc.) for outpatient wound care;
4. description of the postoperative course, specifying presence or abscence of any known complications, i.e. hemorrhage, dyspnea, dysphagia, dysphonia, hypocalcemia, etc.;
5. pharmacological treatments administered to the patient and final pathology report, if available;
6. recommended diet and daily activity exercise;
7. medications on discharge, clearly indicating posology and administration way; if necessary specify thyroid hormone and calcium supplementation;
8. education on how to promptly recognize hypocalcemia-related symptoms;
9. recommended and/or scheduled clinical and/or diagnostic follow-up, as well as eventual radio-iodine treatment in case of cancer;
10. follow-up ENT/phoniatrics, in case of dysphonia and/or rehabilitation program if altered vocal fold mobility is documented.
11. Following thyroidectomy L-thyroxine should be started at a daily dose appropriate for the patient’s weight (0.8 mcg/lb or 1.7 mcg/kg), and serum TSH measured 6–8 weeks postoperatively. Following thyroidectomy, specifically indicated for hyperthyroidism, a specific monitoring of calcium levels must be undertaken and calcium and calcitriol supplementation administered based on biochemical results.

## Outpatient care and follow‑up

The Endocrine Surgery outpatient clinic should be the referral centre for first assessment of patients, either sent for consultation by the primary care physician, the endocrinologist or as a result of histopathological findings.

If during the consultation the final judgement is admission to hospital care, detailed information regarding treatments options should be provided, preferably in the form of a leaflet to allow the patient to elaborate further. These would consist of:

I. Surgical indications and possible alternative medical treatments;
II. Advantages of the surgical treatment over alternative therapy and risks related to the intervention in the short and long-term.

At the end of the consultation, it is good practice to summarise the outcome in a medical report including the following:

- General medical condition, comprehensive of past and present medical history, or any condition potentially requiring special care;
- physical examination;
- diagnosis;
- proposed treatment;
- additional laboratory test and/or imaging (if necessary);
- agreement or disagreement with other opinions from colleagues who previously assessed the same patient (if any report is provided by the patient during the consultation);
- postoperative fiberoptic laryngoscopy is mandatory if dysphonia appears, and eventually followed by speech therapy, according to ENT’ and/or phoniatrist’s judgement.
- In the follow up endocrinological consultation is recommended.

For patient with thyroid carcinoma a multidisciplinary approach [168] including the surgeon, the endocrinologist, the nuclear medicine physician, the pathologist and the radiology, the molecular biologist and the radiation oncologist, is recommended to provide a comprehensive and multimodal management of the disease, as diagnosis and treatment could be complementary in different phases and patients, especially in complex cases [169].

On the basis of cancer risk recurrence, in patients with differentiated carcinoma, postoperative radioiodine therapy indication [170] may be posed, therefore risk stratification appears essential, in fact controversy for its use still remains in intermediate-risk and some low-risk patients to establish the most appropriate thyrosuppressive therapy, as well as intensity and frequency of follow-up.

Depending on the clinical course of disease and response to therapy, the risk of recurrence and mortality may vary over time. Reclassification of risk based on the information obtained during follow-up is valuable, and it is essential to ensure proper management. Dynamic risk assessment should be used to guide all aspects of thyroid cancer management, from the beginning, before a definitive diagnosis is made, and continuing through the final follow-up visit [171].

Registry data are crucial in defining good practice in patient evaluation, benchmarking, and improving the overall quality of the process; they also allow transparency of practices and provide an easy reference for future comprehensive assessment of long-term cancer risk, therefore the availability of a database that can be searched and updated by the different teams is highly desirable [172].

## Data Availability

Not available.
